# Amphiphilic Molecules, Interfaces and Colloids—2nd Edition

**DOI:** 10.3390/molecules31122034

**Published:** 2026-06-10

**Authors:** Plamen Tchoukov, Khristo Khristov

**Affiliations:** 1 Institute of Physical Chemistry, Bulgarian Academy of Sciences, “Acad. Georgi Bonchev” Str. Bl. 11, 1113 Sofia, Bulgaria; 2 National Centre of Excellence Mechatronics and Clean Technologies, 8 bul. Kliment Ohridski, 1756 Sofia, Bulgaria

## 1. Introduction

The relationship between the chemical structure and properties of amphiphilic molecules, their adsorption behavior and organization at interfaces, and the properties of colloidal systems are a primary research area for colloid chemistry, chemical engineering, and a wide range of industries, such as pharmaceuticals, food, resource extraction, and many others [1,2,3]. Studying the molecular structure–interfacial behavior relationships is also essential for understanding processes in living matter and the environment. The design of colloidal systems with the desired functionalities requires both knowledge of the interactions at the nano- and micro-scale level, and the thermodynamic state of the systems as a whole. In many processes of practical interest, the colloidal systems are far from equilibrium and dynamic properties, transient states, transport phenomena, and hydrodynamics are of interest. Besides the properties of interfaces, the colloidal stability is also governed by surface forces and drainage of thin liquid films between dispersed colloidal particles, which is strongly influenced by the mobility of the interfaces and Marangoni flows [4]. A wide range of experimental techniques, including interfacial, surface-force, optical, scattering, spectroscopic, and rheological, are typically employed to study different aspects of these complex systems.

There is increasing interest in the application of bio-based, sustainable and biodegradable amphiphilic molecules driven by increasing environmental awareness and new regulatory requirements [5]. Implementation of these new alternative chemistries, preferably derived from sustainable and renewable resources, requires extensive analytical and physicochemical characterization of their structure, behavior at the interfaces and in the bulk solutions.

Recently, it has become increasingly clear that the functionality of amphiphilic molecules is not determined solely by classical descriptors such as hydrophilic–lipophilic balance (HLB), critical micelle concertation (CMC), or packing parameters. Instead, their behavior is governed by the collective system free energy, which controls molecular self-assembly across multiple length and time scales. Computer simulations techniques like Molecular Dynamics (MD), Density Functional Theory (DFT), Dissipative Particle Dynamics (DPD), and AI-assisted generative models now make it possible to connect molecular architecture directly to properties such as adsorption, micellization, vesicle formation, interfacial rheology, thin-film stability, and transport properties.

This Reprint contains the collection of papers published in the Second Edition of the Special Issue of *Molecules* titled “Amphiphilic Molecules, Interfaces and Colloids”. The presented collection includes research articles ranging from theoretical aspects of ionic effects on surface forces in thin liquid films, new green sustainable surfactants, and insights into the relationship of molecular structure–interfacial–colloidal properties, to optimizations of cosmetic products and new testing procedures.

## 2. An Overview of the Contributions

Blanco et al. (Contribution 1) investigated the stereoisomeric effects of diammonium-cyclohexane (DACH) counterions on the self-assembly of amino acid-based surfactants (AAABs). Four AABSs derived from undecanoyl glycine, L-alanine, L-valine, and L-leucine were combined with six DACH counterions corresponding to the cis and trans isomers of 1,2-, 1,3-, and 1,4-DACH. Critical micelle concentrations and micelle sizes were determined by conductimetry and dynamic light scattering. The counterion binding degrees (β) were calculated to assess micelle stability and geometry-optimized DACH isomer structures were obtained using density functional theory (DFT). The authors found that, although the self-assembly tendencies followed the expected trend with increasing surfactant hydrophobicity, the trans-1,3-DACH counterion disrupted this trend, leading to higher CMCs values that were largely independent of the AABS side chain. The results highlight the important role of counterion structure in determining AABS behavior.

Zapolski et al. (Contribution 2) reported a thought-provoking correlation between HLB values of series of sorbitan esters (sorbitan monopalmitate, sorbitan tristearate, sorbitan monooleate, and sorbitan sesquioleate) and the parameters of the π–A Langmuir isotherm of their monolayers at water surface. The ratio between the slopes corresponding to the first and second stages of monolayer compression is determined and plotted against the calculated HLB values of the surfactants. A strong linear relationship is observed, with a coefficient of correlation 0.95.

Slavova et al. (Contribution 3) developed in vitro procedures using optical methods, artificial skin and model soils (sebum and dimethicone) for evaluation of the cleansing efficacy of formulations with applications in personal care. The authors conducted a systematic study on the soil removal mechanisms of formulations based on widely used in personal care surfactants disodium laureth sulfosuccinate (DSLSS), sodium laureth sulfate (SLES), sodium dodecyl sulfate (SDS), dodecyl trimethyl ammonium bromide (DTAB), and coco glucoside (CG). Nonionic CG removed dimethicone at a lower concentration than ionic surfactants. Combining CG with ionic surfactants improved cleaning at lower total concentrations. Two main mechanisms were outlined: emulsification and roll-up. The reported results have the potential for a direct application in product evaluation and the development process in the cosmetic industry.

Kowalczuk et al. (Contribution 4) reported positive synergetic effects of trisodium citrate (TSC) and silver nanoparticles (AgNPs) on the self-assembly of alanine derivative gelator (C12Ala) hydrogels. Thermal analysis (TGA/DSC), high-resolution nuclear magnetic resonance spectroscopy (HR-MAS NMR), and optical microscopy provided insights into the interactions and supramolecular assemblies of the studied three-component system. AgNPs alone disturb the C12Ala self-assembled structures; however, the presence of TSC molecules shields the gelator molecules from electrostatics interactions with the nanoparticles, allowing for the formation of stable hydrogels.

Mendoza et al. (Contribution 5) explored the application of self-microemulsifying delivery systems (SMEDS) formulated with a surfactant blend of lecithin (Le) and polyglycerol-10-caprylate (PG10C), which was mixed with ethyl oleate as an oil phase. This SMEDS was diluted with 0.9% *w*/*v* NaCl aqueous solution at different ratios and evaluated as a preservation media for dry instant yeast (Saccharomyces cerevisiae). The authors applied the hydrophilic–lipophilic difference and net-average curvature (HLD–NAC) model (available as an Excel-based tool) to predict the phase behavior of diluted SMEDS formulations.

Dimitrova and Slavchov (Contribution 6) studied surface forces in thin liquid films (emulsion and foam films) stabilized by ionic surfactants and draining in a charge regulation regime, assuming the Davies isotherm charge regulation condition. The authors concluded that the thinning of a foam film can trigger a surface phase transition from a 2D liquid expanded state to a 2D gaseous state. This phase transition coincides with a simultaneous jump in the film thickness and the two different thickness states of the film could coexist in equilibrium. These theoretical predictions might serve as a guide for future experiments to confirm these effects.

Thanh-Blicharz et al. (Contribution 7) reported that the chemical and physical modification of starch leads to changes in the molecular structure, including an increase in molecular weight and the degree of branching, which in turn affect its to adsorption behavior at the water/oil interface, rheological properties, and consequently its ability to stabilize emulsions.

Suzuki et al. (Contribution 8) investigated nanoparticles (NPs) of naturally occurring food colorants (β-carotene, lycopene, astaxanthin, and lutein) prepared through a reprecipitation method. The study focused on understanding the role of the solvent used during reprecipitation for the molecular self-assembly and resulting properties of the nanoparticles. The authors found that the terminal groups of carotenoids significantly influence the intermolecular interactions, thereby altering the structural and optical properties of the NPs. These findings are expected to contribute to the development of new technologies for controlling the color properties of carotenoids, which are of considerable interest for food industry.
